# Frequencies of SF3B1, NOTCH1, MYD88, BIRC3 and IGHV mutations and TP53 disruptions in Chinese with chronic lymphocytic leukemia: disparities with Europeans

**DOI:** 10.18632/oncotarget.3101

**Published:** 2014-12-31

**Authors:** Yi Xia, Lei Fan, Li Wang, Robert Peter Gale, Man Wang, Tian Tian, Wei Wu, Liang Yu, Yao-Yu Chen, Wei Xu, Jian-Yong Li

**Affiliations:** ^1^ Department of Hematology, The First Affiliated Hospital of Nanjing Medical University, Jiangsu Province Hospital, Nanjing, China; ^2^ Haematology Research Centre, Division of Experimental Medicine, Department of Medicine, Imperial College London, London, United Kingdom; ^3^ Department of Hematology, Huai'an First People's Hospital, Nanjing Medical University, Huai'an, Jiangsu Province, China

**Keywords:** SF3B1, NOTCH1, MYD88, mutation, chronic lymphocytic leukemia

## Abstract

We studied 307 consecutive Chinese with chronic lymphocytic leukemia (CLL) in diverse disease-stages before and after diverse therapies for mutations in several CLL-related genes. Mutation frequencies were *SF3B1,* 5%*, NOTCH1,* 8%*, MYD88,* 8%, *BIRC3*, 2%, *TP53,* 15% and *IGHV*, 60%. Several of these frequencies differ from those reported in persons of predominately European descent with CLL. Biological and clinical associations were detected including *SF3B1* and *NOTCH1* mutations with un-mutated *IGHV*, *MYD88* mutations with mutated *IGHV*, *SF3B1* mutations with fludarabine-resistant CLL and *NOTCH1* mutation with advanced Binet disease stage and with +12. The *NOTCH1* correlation with briefer survival was confirmed in multivariate analyses but the *SF3B1* correlation was confounded by concurrent mutations in *TP53* and germline *IGHV*. We show differences in incidence and prognostic impact of mutations in Chinese and CLL compared with persons of predominately European descent with CLL. These data may give insights into the etiology and biology of CLL and suggests different risk stratification models may be needed for different CLL populations.

## INTRODUCTION

Biology and clinical course of chronic lymphocytic leukemia (CLL) are heterogeneous. An unfavorable prognosis is associated with certain cytogenetic and molecular abnormalities such as del(11q22-q23), *TP53* disruption and un-mutated immunoglobulin heavy-chain variable region (*IGHV*) state [1-3]. Mutations in RNA-splicing and processing, genes involved in Notch signaling, inflammatory pathway genes and genes in the Wnt pathway are also associated with briefer survival of persons with CLL [4].

Mutations in *SF3B1*, *NOTCH1*, *MYD88* and *BIRC3* are relatively rare in persons of predominately European descent with CLL with frequencies of 5-18%, 8-12%, ~3%, and ~2% [4-10]. However, mutations of these genes in a sub-clone of CLL cells undetectable by conventional sequencing may become detectable as CLL progresses. It is also likely some new mutations are acquired as CLL progresses with or without therapy [11]. We studied frequencies and prognostic associations of mutations of *SF3B1*, *NOTCH1, MYD88, BIRC3, TP53* and IGHV and cytogenetic abnormalities in 307 Chinese with CLL.

## RESULTS

### Subjects

Characteristics of the 307 subjects are summarized in Table 1. Median age was 61 years (range, 16- 92 years) with a male/female ratio of 2:1. Median follow-up is 35 months (range, 1-267 months),

**Table 1:** Clinical characteristics

	N (%)
Sex (n=307)	
Male	204 (66)
Female	103 (34)
Age (n=307)	
≥60	164 (53)
<60	143 (47)
Binet (n=297)	
A	129 (43)
B	70 (24)
C	98 (33)
Disease States (n=307)	
At diagnosis	166 (54)
Progressive	88 (29)
Relapsed	25 (8)
Refractory	28 (9)

### *SF3B1, NOTCH1, MYD88, BIRC3* and *TP53* mutations

307 subjects were studied for *SF3B1* mutations. 15 (5%) had a *SF3B1* mutation. All mutations were missense with p.K700E the most frequent (N=10). 24 of 295 (8%) of subjects analyzed had *NOTCH1* mutations of whom 17 had c.7544-7545delCT. Other mutations were nonsense (N=3) or out-of-frame (N=4). Two mutations, p.L2049fs*1 and p.Q2404X, were not reported by COSMIC or other studies. Mutations in exons 3-5 of *MYD88* were detected in 23 (8%) of 295 subjects analyzed. The most frequent mutation was p.L265P (N=15). p.V271F is a new mutation. 5 of 238 (2%) had a *BIRC3* mutation including 4 out-of-frame and 1 in-frame. 47 (15%) of 307 subjects analyzed had *TP53* mutations. Most mutations were in the DNA-binding domain (N=45; 94%).

Subjects with *SF3B1* mutation were more likely to have concomitant *TP53* mutation (5 of 15 *vs*. 42 of 292; *P*=0.047). Also, *MYD88* mutations were exclusive of *NOTCH1* and *SF3B1* mutations. Detailed data are presented in Supplement Table 1.

### IGHV Mutations

In 118 of 299 (40%) subjects *IGHV* was germline. The most frequently used *IGHV* genes were *IGHV*4-34 (N=38; 13%), *IGHV*3-23 (N=33; 11%), *IGHV*3-7 (N=30, 10%), *IGHV*4-39 (N=21; 7%), *IGHV*4-59 N=15; 5%), *IGHV*1-69 (n=15; 5%) and *IGHV*3-21 (N=9; 3%).

**Table 2:** Mutation frequency by disease state

	Diagnosis	Progressive	Relapsed	Refractory
*SF3B1*	6/166 (4%)	4/88 (5%)	0/25	5/28 (18%)
*NOTCH1*	4/158 (3%)	9/84 (11%)	4/25 (16%)	7/28 (25%)
*MYD88*	15/158 (10%)	7/84 (8%)	1/25 (4%)	0/28
*BIRC3*	2/119 (2%)	1/71 (1.4%)	2/21 (10%)	0/27
*TP53*	13/166 (8%)	18/84 (21%)	4/25 (16%)	12/28 (43%)

### Clinical correlations

*NOTCH1* mutations were detected more frequently in subjects with advanced Binet stages (stage-A, 4 of 126; stage-B: 6 of 66; stage-C, 14 of 94; *P*=0.008). *TP53* mutations were also more common in subjects with advanced Binet stage (stage-A, 11 of 129; stage-B,12 of 70; stage-C, 23 of 98; *P*=0.008). We found no significant correlation between Binet stage and *SF3B1* or *MYD88* mutations (Table 3) but power to detect an association is limited because of the low frequency of mutations. *MYD88* mutations were less frequent in subjects in whom CD38 was ≥30% (1 of 67 *vs*, 22 of 223; *P*=0.026). *NOTCH1* mutations were associated with CD38 ≥30% but this difference was not significant (9 of 67 *vs.* 15 of 223; *P*=0.081). We found no correlation between *SF3B1* or *TP53* mutations and CD38 ≥30%. There was also no correlation between any of the mutations we studied and ZAP70 ≥20%. *BIRC3* mutations were excluded from this analysis because they were so rare (Table 3).

*SF3B1*, *NOTCH1* and *TP53* mutations were significantly correlated with germline *IGHV* (*SF3B1,* 10 of 118 *vs.* 5 of 181; *P*=0.027; *NOTCH1*, 21 of 115 *vs.* 2 of 170; *P*<0.001; *TP53,* 28 of 118 *vs.* 19 of 181: *P*=0.002). In contrast, *MYD88* mutations were more common in subjects with mutated *IGHV* (2 of 115 *vs.* 21 of 172; *P*=0.001; Table 3). *NOTCH1* mutations were especially common in subjects using *IGHV*4-39 (4 of 19 *vs.* 19 of 268; *P*=0.03) and *IGHV*1-69 (6 of 15 *vs.* 17 of 255; *P*<0.001). *SF3B1* mutations were correlated with *IGHV*4-59 (4 of 15 *vs.* 11 of 284; *P*=0.004).

**Table 3:** Associations between subject variables and mutations

		SF3B1mut	***P***	NOTCH1mut	***P***	MYD88mut	***P***	TP53mut	***P***
N	307	15/307 (5%)	-	24/295 (8%)	-	19/229 (8%)	-	47/307 (15%)	-
Female	103 (34%)	2/103 (2%)	0.089	7/101 (7%)	0.585	6/101 (6%)	0.391	21/103 (20%)	0.093
Male	204 (66%)	13/204 (6%)		17/194 (9%)		17/194 (9%)		26/204 (13%)	
Age	61	61(±12)	0.911	64(±11)	0.212	62(±8)	0.631	61(±13)	0.890
Binet A	129(43%)	5/129 (4%)	0.305	4/126 (3%)	**0.008**	9/126 (7%)	0.680	11/129 (9%)	**0.008**
Binet B	70 (24%)	6/70 (9%)		6/66 (9%)		4/66 (6%)		12/70 (17%)	
Binet C	98 (33%)	4/98 (4%)		14/94 (15%)		9/94 (10%)		23/98 (24%)	
CD38≥30%	71/302 (24%)	5/71 (7%)	0.356	9/67 (13%)	0.081	1/67 (2%)	**0.026**	10/71 (14%)	0.677
CD38<30%	231/302 (76%)	10/231 (4%)		15/223 (7%)		22/223 (10%)		36/231 (16%)	
ZAP70≥20%	110/272 (40%)	7/110 (6%)	0.613	11/106 (10%)	0.246	6/106 (6%)	0.523	17/110 (16%)	0.774
ZAP70<20%	162/272 (60%)	8/162 (5%)		10/156 (6%)		12/156 (8%)		23/162 (14%)	
*IGHV* M	181/299 (61%)	5/181 (3%)	**0.027**	2/172 (1%,)	**<0.001**	21/172 (12%)	**0.001**	19/181 (11%)	**0.001**
*IGHV* UM	118/299 (40%)	10/118 (9%)		21/115 (18%)		2/115 (2%)		28/118 (24%)	

### Association with cytogenetic abnormalities

**Figure 1:**
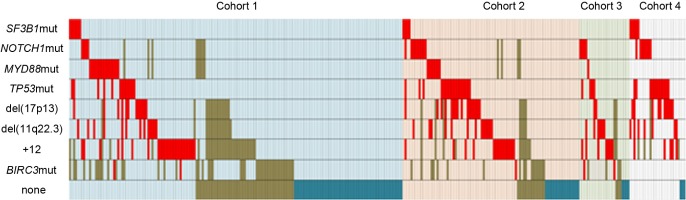
Relationship between *TP53, SF3B1, NOTCH1* and *MYD88* mutations, and cytogenetic abnormalities in 307 subjects with data Cases which presented with a cytogenetic aberration or a mutation are colored in red. Cohort1: Newly-diagnosed; cohort 2: Progressive; no therapy; cohort 3:Relapsed CLL after therapy; and cohort 4: Refractory, therapy-resistant. Subjects are highlighted in light blue, pink, light green and light purple, respectively. Brown means not analyzed.

Cytogenetic abnormalities were common in our subjects including +12 in 59 of 257 (23%), del(17p13) in 42 of 281 (15%) and del(11q22.3) in 40 of 283 (14%). As expected, *NOTCH1* mutations were significantly associated with +12 (9 of 56 *vs.* 12 of 192; *P*=0.020) and *TP53* mutations with del(17p13) (23 of 40 *vs.* 22 of 243; *P*<0.001). Among the 64 subjects with *TP53* abnormalities, 17 had only del(17p13), 23 had *TP53* mutation and del(17p13) and 22 only *TP53* mutation (2 missed del(17p13) data, but got *TP53* mutation). Surprisingly, there was no correlation between *SF3B1* mutations and del(11q22.3) (4 of42 *vs.* 11 of 239; *P*=0.191). *MYD88* mutations did not correlate with cytogenetic abnormalities (Supplement Figure 1). Detailed distribution of mutations and cytogenetic lesions are shown in Figure 1.

### Prognostic relevance of mutations and cytogenetic abnormalities

We divided subjects into 4 prognostic cohorts: (1) newly-diagnosed; (2) progressive; no therapy; (3) relapsed CLL after therapy; and (4) refractory, fludarabine-resistant. Frequency of gene mutations in each cohort is shown in Table 2. Mutation rate of *SF3B1* was significantly higher in subjects in cohort 4 (*P*=0.007). *NOTCH1* was less frequently mutated in subjects in cohort 1 (*P*<0.001) but similar in cohorts 2-4 (*P*>0.05). *TP53* mutation was less frequent in subjects in cohort 1 (*P*=0.004) and more common in subjects in cohort 4 (*P*=0.033). *MYD88* mutation was more common in subjects in cohort 1 and 2 (*P*<0.001). There was no significant association of *BIRC3* mutation with any cohort.

**Figure 2:**
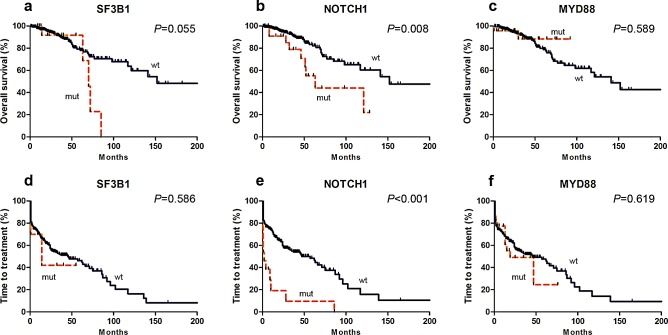
Kaplan-Meier curves of survival (a-c) and time-to-treatment (TTT) (d-f) for *SF3B1* (a and d), *NOTCH1* (b and e) and *MYD88* mutations (c and f)

Prognostic impact of the abnormalities we studied is shown in Figure 2. Survival was significantly briefer in subjects with *NOTCH1* mutation (median, 63 *vs.* 153 months; *P*=0.008), del(11q22.3) (median, 77 *vs.* 152 months; *P*=0.030) and *TP53* disruptions (median, 71 months *vs.* NR, *P*<0.001). *MYD88* mutations had no significant impact on survival (median, NR *vs.* 142 months; *P*=0.590). However, *SF3B1* mutations had only a borderline impact on survival (median, 70 *vs.* 152 months; *P*=0.055) even when 6 subjects with concurrent *TP53* mutations were included. The frequency of *BIRC3* mutations in *BIRC3* was too low for statistical comparisons (Supplement Table 2 ).

**Figure 3:**
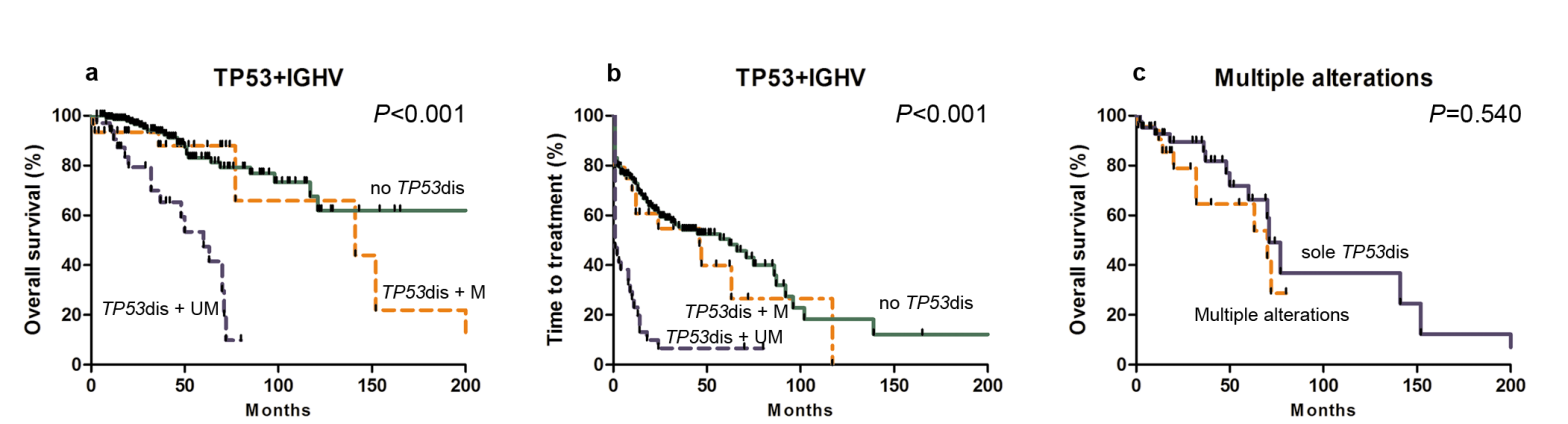
Kaplan-Meier curves of survival (a) and TTT (b) for *TP53* disruption cases with different *IGHV gene*mutation states Comparison of survival subjects with multiple unfavorable alterations including *TP53 vs.* those with *TP53* only mutation is shown in (c).

We divided the *TP53* mutation cohort into 2 cohorts based on *IGHV* rearrangement. 30 of 64 subjects with *TP53* disruption and mutated *IGHV* had longer survival compared with those with *TP53* disruption and germline *IGHV* (median, 141 *vs.* 60 months; *P*=0.001). Survival of the favorable cohort was not significantly different from subjects without *TP53* disruptions (median, 141 months *vs.* NR; *P*=0.308; Figure 3a). There was also no significant survival difference with only *TP53* mutation, only del(17p13) or both (*P*=0.474).

Next, we compared whether subjects with *TP53* disruptions and other unfavorable abnormalities including del(11q22.3) or mutations in *NOTCH1* and/or *SF3B1* mutations had shorter survival compared with those with *TP53* disruptions only. There was no significant difference (median, 70 *vs.* 71 months; *P*=0.540; Figure 3c). Multiple unfavorable abnormalities were more common in subjects with refractory, fludarabine-resistant disease (cohort 4; 6 of 143 *vs.* 14 of 127; *P*=0.033; Supplement Table 3).

The impact of each mutation on TTT was studied in previously untreated subjects (cohort 1 and 2; Table 4). Subjects with *NOTCH1* mutations and/or *TP53* disruptions had briefer TTT than subjects without these abnormalities (median, 2 *vs.* 57 months; *P*<0.001 and 12 *vs.* 62 months; *P*=0.003). Subjects with *TP53* disruptions and mutated *IGHV* had similar TTT as subjects without *TP53* disruptions (median: 46 *vs.* 62 months; *P*=0.423). There was no significant correlation between mutations in *SF3B1* or *MYD88* mutation and TTT (median, 14 *vs.* 46 months; *P*=0.586; median, 19 *vs.* 46 months; *P*=0.619; Figure 2 and 3b).

In multivariate analyses variables independently-correlated with TTT included germline *IGHV* (HR=2.30, 95% CI: 1.51–3.49; *P*<0.001), *TP53* disruptions (HR=1.85, 95% CI: 1.20–2.83; *P*=0.005) and *NOTCH1* mutation (HR=2.17, 95% CI: 1.11–4.23; *P*=0.024).

**Table 4:** Associations between mutations, cytogenetics, time-to-treatment and survival

	Time-to-treatment			Survival		
Variable	N	Median (mo)	P-value	N	Median (mo)	P-value
*TP53* disruption	234		0.003	285		<0.001
Yes	44	12		64	71	
No	190	62		221	NR	
del(11q22.3)	232		0.061	281		0.030
Yes	28	18		42	77	
No	204	47		239	152	
+12	211		0.393	257		0.366
Yes	44	37		59	117	
No	167	66		198	152	
*SF3B1*	254		0.586	307		0.055
Mutated	10	14		15	72	
Wild-type	244	46		292	152	
*NOTCH1*	242		<0.001	295		0.008
Mutated	13	2		24	63	
Wild-type	229	57		271	152	
*MYD88*	242		0.619	295		0.589
Mutated	22	19		23	NR	
Wild-type	220	46		272	141	

## DISCUSSION

We provide data of frequencies of mutations in *SF3B1*, *NOTCH1*, *MYD88, BIRC3* in Chinese with CLL along with data on previously described prognostic variables including *TP53* disruptions*, IGHV* mutation and cytogenetic abnormalities. One striking difference was the frequency, biological features and prognostic impact of *SF3B1* mutations in Chinese with CLL. The frequency we detected is considerably lower than reported in persons of predominately European descent (Table 2) [4, 7, 8, 12-14]. This low rate might be accounted for by the relatively low sensitivity of the Sanger sequencing we used. For example, Jeromin *et al.* reported about 10% *SF3B1* mutations occurred at a mutation load ≤10%, a level detectable only with next generation sequencing (NGS) and would have been missed by us [13]. However, this is unlikely to explain the disparate frequencies we observed. We found *SF3B1* mutation frequency was significantly higher in subjects with advanced, fludarabine-resistant CLL consistent with the notion detection of these mutations result from expansion of a sub-clone of CLL cells and are acquired during disease progression [15, 16].

We also found discrepancies regarding to *IGHV* gene use by *SF3B1* mutated cases between our subjects and persons of predominately European descent. Strefford *et al*. reported a much higher frequency of *SF3B1* mutations in stereotyped *IGHV*3-21 [17]. A recent study of 1160 untreated persons of predominately European descent with CLL reported frequent *SF3B1* mutations in subjects with *IGHV*3-21 and *IGHV*1-69 gene use [13]. Many of our subjects had germline *IGHV*. In contrast, *SF3B1* mutations were not correlated with *IGHV*1-69 or *IGHV*3-21 but rather with *IGHV*4-59. This low frequency of *IGHV*1-69 use in Chinese with CLL is previously reported [18, 19]. Only 9 of 299 subjects had *IGHV*3-21 use and only 15 used *IGHV*1-69. Furthermore, only one *IGHV*1-69 user had *SF3B1* p.K700E mutation. The reason for the disparities between Chinese and persons of European descent with CLL are unknown and could relate to the low incidence of *SF3B1* mutations and *IGHV*1-69 and *IGHV*3-21 use in Chinese with CLL. It is also possible different antigenic stimuli operate in diverse geographies [20]. Interestingly, *SF3B1* mutation was not associated with advanced Binet stage, CD38 ≥30% or del(11q22.3) but was correlated with *TP53* mutation. These data suggest distinct biological features of *SF3B1* mutated cases of CLL in Chinese may differ from those in persons of predominately European decent.

Incidence of *NOTCH1* mutations in our cohort is relatively low at diagnosis but increased with disease progression. These data are compatible with a sub-clone of CLL cells below the sensitivity of Sanger sequencing followed by clonal expansion during disease course, acquisition of additional mutations spontaneously for as a consequence of therapy or both [11, 16]. Mutation detection methods with higher sensitivity, such as allele specific PCR, should be able to distinguish these possibilities which are not mutually-exclusive [21]. The hot spot c.7544-7545delCT deletion accounted for about 70% of our mutated cases. Notably, two novel nonsense mutations were found suggesting the possibility of novel *NOTCH1* mutation sites. Significant associations were observed between *NOTCH1* mutations and advanced Binet stage, germline *IGHV* and +12. *IGHV*4-39 and *IGHV*1-69 were frequently used in subjects with *NOTCH1* mutations. +12 and *NOTCH1*mutations are reported to characterize *IGHV*4-39 CLL belonging to subset 8 and with higher risk of transformation to diffuse large B-cell lymphoma (DLBCL; Richter syndrome) [22, 23]. We observed only 1 case of transformation, possibly because of brief follow-up.

Another interesting finding is the high frequency of *MYD88* mutation in our cohort [12-14]. *MYD88* mutation is common in subjects with mutated *IGHV* and rare in the CD38 ≥30% cohort. Moreover, almost all *MYD88* mutations occurred in untreated subjects consistent with the hypothesis *MYD88* mutation occurs early in CLL development [11]. In addition, we found no significant correlation between *BIRC3* mutation in fludarabine-resistant patients as previously reported [10].

We show *NOTCH1* and *TP53* disruptions are unfavorable factors for survival and TTT consistent with prior studies in persons of predominately European descent [5, 6, 8, 11-14, 16]. In contrast, the prognostic impact of *SF3B1* mutations is modest, if any. The unfavorable impact of *SF3B1* mutation on survival was largely dependent on concurrent mutations in *TP53* and germline *IGHV*. Our data contradict other studies in persons of predominately European descent in whom *SF3B1* mutation is a strong independent predictor of brief survival [4, 7, 8, 10, 11-16]. We had, of course, little power to test the impact of *SF3B1* mutation alone on survival because of the low frequency so our conclusion should be viewed cautiously.

We found high rate of subjects with *TP53* mutations in persons without del(17p13). These subjects had a poor prognosis similar to those with both abnormalities similar to our prior report [24]. However, we also found a cohort of subjects with *TP53* disruptions had stable disease. Subjects with *TP53* disruptions and germline *IGHV* had the worst prognosis whereas those with *TP53* disruptions and mutated *IGHV* gene had longer TTT and survival similar to subjects without *TP53* abnormality. Others report similar data emphasizing the combination of *IGHV* rearrangement and *TP53* disruptions might be a better predictor of prognosis than either variable alone [14, 25].

Recently, Greipp *et al.* reported persons with CLL with del(17p13) and del(11q22.3) had briefer survival compared with persons with del(17p13). These persons were termed *double hit* CLL [26]. Based on this report we compared outcomes of persons with *TP53* disruptions and other unfavorable abnormalities including del(11q22.3) or mutations in *NOTCH1* and/or *SF3B1* mutations with those with *TP53* mutation only. We found no significant difference in outcomes. There are several possible explanations for this disparity including different populations, different *NOTCH1* and *SF3B1* mutations and different cut-off values for FISH in the 2 studies. Nevertheless, our data support the concept many unfavorable genetic abnormalities are more common in advanced CLL.

In multivariate analyses, *NOTCH1* mutation, germline *IGHV* and *TP53* disruptions were independently correlated with TTT. Detection of *NOTCH1* mutations helped us reclassify 13 subjects with no other unfavorable risk factors. Patients with *NOTCH1* and/or *SF3B1* mutations were considered intermediate to high risk in other reports [12-14]. In our cohort *NOTCH1* mutation had the greatest risk of requiring therapy which would also indicate high-risk disease. In contrast, *SF3B1* mutations had little or no prognostic impact and were included in the good-risk cohort. These disparities highlight the need for different risk classifications for different populations with CLL.

In conclusion, we studied frequency and prognostic impact of cytogenetic and molecular abnormalities in a large series of Chinese with CLL and compared these data with data from persons of predominately European descent with CLL. Our study highlights important similarities but also which may offer important clues to the etiology and biology of CLL.

## METHODS

### Subjects

The study cohort was a single-center consecutive series of 307 Chinese with CLL. Subjects were diagnosed from November, 1991 to April, 2014. Subjects provided informed consent according to institutional guidelines. Diagnosis of CLL was based on International Workshop on CLL-National Cancer Institute (IWCLL-NCI) criteria [27].

### Analyses of *SF3B1, NOTCH1, MYD88* mutations and *TP53* disruptions

Genomic DNA was isolated from mononuclear cells using the QIAamp DNA Blood Kits (Qiagen, Düsseldorf, Germany) according to the manufacturer's recommendation. Direct Sanger sequencing was performed for exon 14-16 of *SF3B1*, PEST domain of *NOTCH1*, exon 3-5 of *MYD88*, exon 6-9 of *BIRC3* and exon 4-9 of *TP53*. Primers are listed in Supplement Table 4.

### Cytogenetics

Fluorescence *in situ* hybridization (FISH) analysis was performed on most subjects to detect of del(11q22.3) (n=281), del(17p13) (n=283) and trisomy 12 (n=257). The following fluorescent-labeled probes were used: LSI *ATM* (11q22.3), LSI p53 (17p13) and CEP12 (centromere 12). Probes were purchased from Vysis, Downers Grove, IL, USA. FISH was performed as described [24]. Cut-off levels for positivity were 7.7%, 5.2%, and 3.0% for del(11q22.3), del(17p13) and +12. Although not all subjects with *TP53* mutation were analyzed for del(17p13), we refer the cohort with *TP53* mutation or del(17p13) with no *TP53* mutation testing as *TP53* disruptions.

### Immunophenotyping and *IGHV* mutation analyses

Immunophenotyping of CD38 and ZAP-70 expression and *IGHV* sequencing were performed as described [28]. Positive cut-off values were 30% and 20%. Germline *IGHV* was defined as ≥98% germline homology.

### Statistical analyses

Survival was calculated as time from diagnosis until death or last follow-up. Time-to-treatment (TTT) was calculated as time from diagnosis until first treatment. Calculations were performed using SPSS (version 19.0) software (IBM Corporation, Armonk, NY, USA). Categorical variables were compared using χ^2^ test and continuous variables using Student t-test. Survival curves were constructed by Kaplan-Meier method and log-rank test was used for significant associations. Multivariate analysis was done by Cox proportional hazard regression. Multivariate analysis of TTT was done. However, there were too few events to analyze survival. *P*-values were two-sided; *P*<0.05 was considered significant.

## SUPPLEMENTARY MATERIAL, FIGURES, TABLES


