# Supplementary material for: Frequencies of SF3B1, NOTCH1, MYD88, BIRC3 and IGHV mutations and TP53 disruptions in Chinese with chronic lymphocytic leukemia: disparities with Europeans
Source: Oncotarget. 2014 Dec 31;6(7):5426–34. doi: 10.18632/oncotarget.3101 (PMC4467158; doi:10.18632/oncotarget.3101)
Supplement: Supplementary file 1 [file oncotarget-06-5426-s001.pdf]

Frequencies of SF3B1, NOTCH1, MYD88, BIRC3 and IGHV mutations and *TP53* disruptions in Chinese with chronic lymphocytic leukemia: disparities with Europeans

Supplementary Material

Figure S1

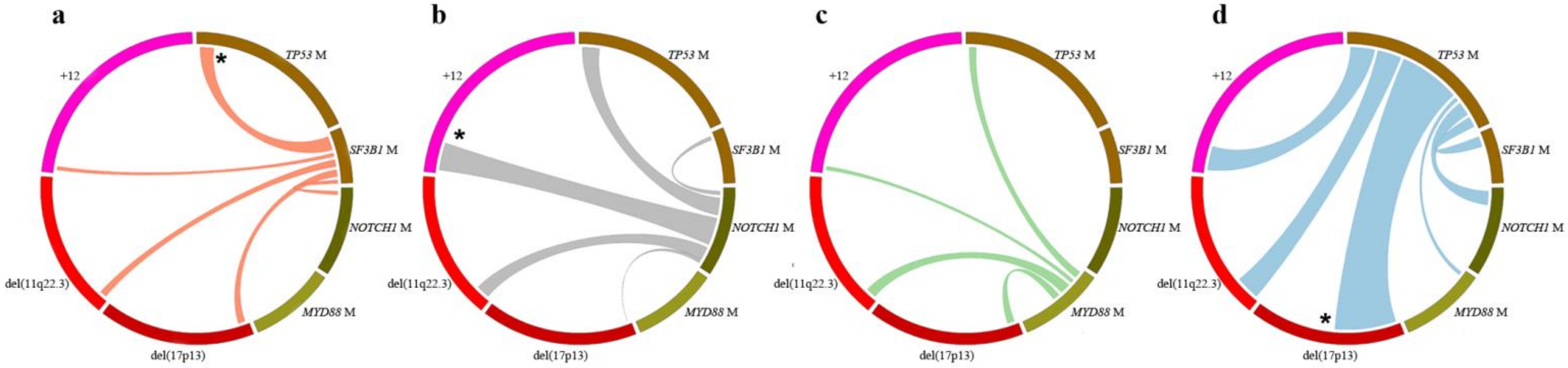

Figure S1: Circos plots showing the pairwise co-occurrence of mutational and cytogenetic lesions in the training series. The length of the arc corresponds to the frequency of the genetic lesions. The width of the ribbon corresponds to the percentage of patients in which the two connected genetic lesions co-occurred. The asterisk indicates statistically significant associations ($p<0.05$).

TableS1: *TP53*, *NOTCH1*, *SF3B1*, *MYD88* and *BIRC3* mutations in the study group

Sample ID	Gene	Nucleotide change	Amino acid change	Refseq
NJ-22	TP53	c.745A>T	p.R249W	NM_000546.5
NJ-31	TP53	c.743G>A	p.R248Q	NM_000546.5
NJ-35	TP53	c.806G>A	p.S269N	NM_000546.5
NJ-39	TP53	c.916C>T	p.R306X	NM_000546.5
NJ-47	TP53	c.487_489delTAC	p.Y163delY	NM_000546.5
NJ-49	TP53	c.578A>G	p.H193R	NM_000546.5
NJ-50	TP53	c.724T>A	p.C242S	NM_000546.5
NJ-55	TP53	c.818G>A	p.R273H	NM_000546.5
NJ-63	TP53	c.403T>C	p.C135R	NM_000546.5
NJ-74	TP53	c.430C>T	p.Q144X	NM_000546.5
NJ-86	TP53	c.536A>G	p.H179R	NM_000546.5
NJ-122	TP53	c.358A>G	p.K120E	NM_000546.5
NJ-131	TP53	c.437G>A	p.W146X	NM_000546.5
NJ-140	TP53	c.674T>G	p.V225G	NM_000546.5
NJ-147	TP53	c.473G>T	p.R158L	NM_000546.5
NJ-164	TP53	c.861G>T	p.E287D	NM_000546.5
NJ-193	TP53	c.772G>A	p.E258K	NM_000546.5
NJ-195	TP53	c.752T>G	p.I251S	NM_000546.5
NJ-203	TP53	c.769C>G	p.L257V	NM_000546.5
NJ-204	TP53	c.493_493del1	p.Q165fs*5	NM_000546.5
NJ-206	TP53	c.883C>G	p.P295A	NM_000546.5
NJ-225	TP53	c.536A>G	p.H179R	NM_000546.5
NJ-270	TP53	c.818G>T	p.R273L	NM_000546.5
NJ-276	TP53	c.652G>A	p.V218M	NM_000546.5

NJ-288	TP53	c.731G>A	p.G244D	NM_000546.5
NJ-298	TP53	c.535C>T	p.H179Y	NM_000546.5
NJ-300	TP53	c.714_715insT	p.N239fs*1	NM_000546.5
NJ-316	TP53	c.478_478del1	p.M160fs*10	NM_000546.5
NJ-344	TP53	c.517G>A	p.V173M	NM_000546.5
NJ-355	TP53	c.430C>T	p.Q144X	NM_000546.5
NJ-357	TP53	c.685_686del2	p.C229fs*10	NM_000546.5
NJ-358	TP53	c.406C>T	p.Q136X	NM_000546.5
NJ-376	TP53	c.159G>T	p.W53C	NM_000546.5
NJ-378	TP53	c.586C>T	p.R196X	NM_000546.5
NJ-384	TP53	c.831T>G	p.C277W	NM_000546.5
NJ-385	TP53	c.747G>T	p.R249S	NM_000546.5
NJ-392	TP53	c.586C>T, c.536A>G	p.R196X，p.H179R	NM_000546.5
NJ-09	TP53	c.814G>T	p.V272L	NM_000546.5
NJ-283	TP53	c.724T>A	p.C242S	NM_000546.5
NJ-25	TP53	c.818G>T	p.R273L	NM_000546.5
NJ-398	TP53	c.844C>T	p.R282W	NM_000546.5
NJ-423	TP53	c.829T>G	p.C277G	NM_000546.5
NJ-433	TP53	c.358A>G	p.K120E	NM_000546.5
NJ-439	TP53	c.206C>G	p.A69G	NM_000546.5
NJ-440	TP53	c.829T>G	p.C277G	NM_000546.5
NJ-444	TP53	c.829T>G	p.C277G	NM_000546.5
NJ-448	TP53	c.733G>A	p.G245S	NM_000546.5
NJ-15	SF3B1	c.2225G>A	p.G742D	NM_012433.2
NJ-21	SF3B1	c.2098A>G	p.K700E	NM_012433.2
NJ-45	SF3B1	c.1877A>T	p.N626I	NM_012433.2

NJ-47	SF3B1	c.2098A>G	p.K700E	NM_012433.2
NJ-74	SF3B1	c.1866G>T	p.E622D	NM_012433.2
NJ-98	SF3B1	c.2098A>G	p.K700E	NM_012433.2
NJ-140	SF3B1	c.2098A>G	p.K700E	NM_012433.2
NJ-147	SF3B1	c.1996A>G	p.K666E	NM_012433.2
NJ-175	SF3B1	c.2098A>G	p.K700E	NM_012433.2
NJ-203	SF3B1	c.2098A>G	p.K700E	NM_012433.2
NJ-273	SF3B1	c.2098A>G	p.K700E	NM_012433.2
NJ-307	SF3B1	c.2098A>G	p.K700E	NM_012433.2
NJ-314	SF3B1	c.2225G>A	p.G742D	NM_012433.2
NJ-371	SF3B1	c.2098A>G	p.K700E	NM_012433.2
NJ-372	SF3B1	c.2098A>G	p.K700E	NM_012433.2
NJ-24	NOTCH1	c.7544_7545delCT	p.P2515fs*4	NM_017617.2
NJ-48	NOTCH1	c.7210C>T	Q2404X	NM_017617.2
NJ-74	NOTCH1	c.7544_7545delCT	p.P2515fs*4	NM_017617.2
NJ-76	NOTCH1	c.7544_7545delCT	p.P2515fs*4	NM_017617.2
NJ-90	NOTCH1	c.7544_7545delCT	p.P2515fs*4	NM_017617.2
NJ-92	NOTCH1	c.7544_7545delCT	p.P2515fs*4	NM_017617.2
NJ-141	NOTCH1	c.7544_7545delCT	p.P2515fs*4	NM_017617.2
NJ-147	NOTCH1	c.7544_7545delCT	p.P2515fs*4	NM_017617.2
NJ-160	NOTCH1	c.7544_7545delCT	p.P2515fs*4	NM_017617.2
NJ-164	NOTCH1	c.7330C>T	Q2444X	NM_017617.2
NJ-174	NOTCH1	c.7535_7536insC	S2513fs*3	NM_017617.2
NJ-191	NOTCH1	c.7375C>T	Q2459X	NM_017617.2
NJ-195	NOTCH1	c.7544_7545delCT	p.P2515fs*4	NM_017617.2
NJ-207	NOTCH1	c.7544_7545delCT	p.P2515fs*4	NM_017617.2

NJ-216	NOTCH1	c.7544_7545delCT	p.P2515fs*4	NM_017617.2
NJ-220	NOTCH1	c.7222delC	p. L2049fs*1	NM_017617.2
NJ-267	NOTCH1	c.7544_7545delCT	p.P2515fs*4	NM_017617.2
NJ-346	NOTCH1	c.7544_7545delCT	p.P2515fs*4	NM_017617.2
NJ-348	NOTCH1	c.7544_7545delCT	p.P2515fs*4	NM_017617.2
NJ-10	NOTCH1	c.7357_7358insCG	p.V2453fs*25	NM_017617.2
NJ-435	NOTCH1	c.7544_7545delCT	p.P2515fs*4	NM_017617.2
NJ-439	NOTCH1	c.7544_7545delCT	p.P2515fs*4	NM_017617.2
NJ-446	NOTCH1	c.7443delC	p.L2482fs*1	NM_017617.2
NJ-448	NOTCH1	c.7544_7545delCT	p.P2515fs*4	NM_017617.2
NJ-17	MYD88	c.794T>C	p.L265P	NM_002468.4
NJ-70	MYD88	c.695T>C	p.M232T	NM_002468.4
NJ-78	MYD88	c.794T>C	p.L265P	NM_002468.4
NJ-91	MYD88	c.794T>C	p.L265P	NM_002468.4
NJ-101	MYD88	c.794T>C	p.L265P	NM_002468.4
NJ-120	MYD88	c.794T>C	p.L265P	NM_002468.4
NJ-139	MYD88	c.794T>C	p.L265P	NM_002468.4
NJ-152	MYD88	c.695T>C	p.M232T	NM_002468.4
NJ-177	MYD88	c.794T>C	p.L265P	NM_002468.4
NJ-178	MYD88	c.794T>C	p.L265P	NM_002468.4
NJ-190	MYD88	c.794T>C	p.L265P	NM_002468.4
NJ-206	MYD88	c.656C>G	p.S219C	NM_002468.4
NJ-221	MYD88	c.728G>A	p.S243N	NM_002468.4
NJ-257	MYD88	c.656C>G	p.S219C	NM_002468.4
NJ-294	MYD88	c.794T>C	p.L265P	NM_002468.4
NJ-298	MYD88	c.794T>C	p.L265P	NM_002468.4

NJ-345	MYD88	c.794T>C	p.L625P	NM_002468.4
NJ-352	MYD88	c.794T>C	p.L625P	NM_002468.4
NJ-437	MYD88	c.649G>T	p.V217F	NM_002468.4
NJ-444	MYD88	c.794T>C	p.L265P	NM_002468.4
NJ-440	MYD88	c.794T>C	p.M232T	NM_002468.4
NJ-439	MYD88	c.794T>C	p.L265P	NM_002468.4
NJ-451	MYD88	c.656C>G	p.S219C	NM_002468.4
NJ-158	BIRC3	c.1292_1293 del AA	p. E433fs*3	NM_001165.3
NJ-191	BIRC3	c.1663_1666delAGAA	p.R555fs*12	NM_001165.3
NJ-290	BIRC3	c.1295_1298 del GAGA	p.R432fs*30	NM_001165.3
NJ-305	BIRC3	c.1664_1666 del GAA	p. R555delR	NM_001165.3
NJ-440	BIRC3	c.1295_1298 del GAGA	p.R432fs*30	NM_001165.3

Table S2: Clinical characteristics of patients with *BIRC3* mutation

NO.	Gender	Age	Concomitant alterations	IGHV mutational status	CD38	ZAP-70	Karyotype	Disease status	TTT(mo)	OS(mo)
NJ-158	F	57		UM	92%	76%	Normal	progressive	14	26
NJ-191	F	68	NOTCH1 mutation	UM	87.6%	21.3%	Normal	relapsed	72	121
NJ-290	M	62	+12	M	Negative	Negative	47,XY，+12[8]	at diagnosis	32	35+
NJ-305	M	56		UM	38.50%	74%	NA	relapsed	1	24+
NJ-440	F	71	MYD88 mutation	M	Negative	26.10%	Normal	at diagnosis	76+	76+

Table S3: Clinical characteristics of patients with multiple unfavorable alterations

NO.	Gender	Age	Concomitant alterations	IGHV mutational status	CD38	ZAP-70	Karyotype	Disease status
NJ-22	M	60	del(11q22.3)	UM	Negative	Negative	normal	refractory
NJ-45	M	62	*SF3B1* mutation	UM	32%	28.5%	46,XY,der(9)[2]/46,XY[8]	refractory
NJ-47	F	55	*SF3B1* mutation, del(11q22.3)	UM	52%	Negative	46,XX,der(10),der(11),-17,+mar[10cp]	refractory
NJ-49	M	85	del(11q22.3)	UM	89.6%	Negative	46,XY,i(8q),-11,+12,der(17)t(11;17)(p12;q13)[7]/46,XY[3]	at diagnosis
NJ-55	M	73	del(11q22.3)	UM	Negative	Negative	46,XY,-4,-9,der(9),der(13;17),+mar1,+mar2[2]/46,XY[8]	progressive
NJ-74	M	51	*SF3B1* mutation, *NOTCH1* mutation, del(11q22.3)	UM	49.2%	Negative	46,XY,11q-[2]/46,XY/or/46,XY[20]	refractory
NJ-107	M	50	del(11q22.3)	M	Negative	Negative	normal	at diagnosis
NJ-140	M	78	*SF3B1* mutation	M	Negative	Negative	normal	at diagnosis
NJ-147	F	59	*SF3B1* mutation, *NOTCH1* mutation	UM	Negative	33%	47,XX,+12,-17,+mar[6]	progressive
NJ-164	M	52	*NOTCH1* mutation	UM	98.8%	28%	47,XY,+?12[8]/46,XY[2]	relapsed
NJ-195	F	75	*NOTCH1* mutation	UM	Negative	Negative	normal	progressive
NJ-203	M	47	*SF3B1* mutation	UM	Negative	30.2%	NA	at diagnosis
NJ-225	F	49	del(11q22.3)	UM	Negative	62.20%	normal	progressive
NJ-298	M	73	del(11q22.3)	M	Negative	NA	85,der(X)dic(X;7)(q27;q22)X2,YY,-5,-6,-6,6q+,-7,-7,der(12)t(6;12)X2,-15,-17,der(21)t(17;21)(q10;p13)X2,+21[1]/87,XXYY,-7,-7,-9,-17,-17,21q+X2[1]/46,XY[1]	at diagnosis

NJ-300	M	77	del(11q22.3)	UM	Negative	49.00%	40-42,X,-Y,-1,2p+,-3,3q-,-5,der(7;8)(p10;q10),+der(7),add(11)(p15),-13,17p-,+der(17)add(17)(q25)del(17)(p13),-18,-22,+mar[10cp]	at diagnosis
NJ-389	M	71	del(11q22.3)	UM	Negative	NA	45,XY,add(4)(p16),-17[6]/46,XY,del(13)(q21q32)[2]/46,XY[2]	at diagnosis
NJ-09	M	80	del(11q22.3)	M	Negative	Negative	46,XY,6q-,14q+,17p-[4]/46,idem,5q+[2]P,D,I//43,X,-Y,der(1),t(5;7)(q34;q32),8p-,9p+,15q+,-17,-18,+1(20p)[9]/46,XY[1] D I	relapsed
NJ-398	M	40	del(11q22.3)	UM	Negative	63%	44-46,XY,del(6)(q23),del(11)(q24),del(13)(q14),der(17),18p+,-20,+mar[cp9]/46,XY[1]	progressive
NJ-439	M	44	*NOTCH1* mutation	UM	Negative	Negative	NA	progressive
NJ-448	M	62	*NOTCH1* mutation	UM	Negative	NA	NA	refractory

TableS4: Primer sequences for PCR reaction

Gene	Primer	Sequence	Product length
SF3B1	Exon 14 F	TGACTGTCCTTTCTTTGTTTAC	360bp
	Exon 14 R	ATAGTAAGACCCTGTCTCCTA	
	Exon 15-16 F	TTGGCTGAATAGTTGATATATTGAGAG	553bp
	Exon 15-16 R	AAACACTTTAAAATTCTGTTAGAACCA	
NOTCH1	Exon 34/ PEST F	CAGATGCAGCAGCAGAACCTG	531bp
	Exon 34/ PEST R	AAAGGAAGCCGGGGTCTCGT	

MYD88	Exon 3-5 F	AGCGACATCCAGTTTGTGC	830bp
	Exon 3-5 R	AGGCGAGTCCAGAACCAAG	
BIRC3	Exon 6 F	CCTAATATGTGTTAAATTCTTTGTTCC	328bp
	Exon 6 R	AGACTGATATCAAATCCTTATGAAAAT	
	Exon 7 F	TGGAAGGAAGTTTGTGAGCA	372bp
	Exon 7 R	AAGAGCGTATTTTCAATTGACTTAGA	
	Exon 8 F	TCATAGTAATGCTTTTTCTTTTTCTCC	153bp
	Exon 8 R	AGGCAGTTTGCTTCTTCAGTG	
	Exon 9 F	TGAAGAAGCAAACTGCCTTTTAT	268bp
	Exon 9 R	AAAGTTTAGACGATGTTTTGGTTC	
TP53	Exon 4 F	TGCTCTTTTCACCCATCTAC	353bp
	Exon 4 R	ATACGGCCAGGCATTGAAGT	
	Exon 5-6 F	TGTTCACTTGTGCCCTGACT	467bp
	Exon 5-6 R	TTAACCCCTCCTCCCAGAGA	
	Exon 7 F	GCCTCCCCTGCTTGCCACAG	205bp
	Exon 7 R	GTGCAGGGTGGCAAGTGGCT	
	Exon 8-9 F	AGGGTGGTTGGGAGTAGATG	541bp
	Exon 8-9 R	CAGGAGCCATTGTCTTTGAG	
